# Deciphering Multidrug-Resistant *Pseudomonas aeruginosa*: Mechanistic Insights and Environmental Risks

**DOI:** 10.3390/toxics13040303

**Published:** 2025-04-12

**Authors:** Yang Pei, Péter Hamar, De-Sheng Pei

**Affiliations:** 1 School of Public Health, Chongqing Medical University, Chongqing 400016, China; 2 Chongqing No.11 Middle School, Chongqing 400061, China; 3 Chongqing Miankai Biotechnology Research Institute Co., Ltd., Chongqing 400025, China; 4 Institute of Translational Medicine, Semmelweis University, Tűzoltó Utca 37-47, 1094 Budapest, Hungary

**Keywords:** *Pseudomonas aeruginosa*, multidrug-resistance, CRISPR-Cas9, bridge RNA-guided gene editing, environmental risks

## Abstract

The rise of multidrug-resistant (MDR) *Pseudomonas aeruginosa* (*P. aeruginosa*) presents a significant challenge to clinical treatment and environmental risks. This review delves into the complex mechanisms underlying MDR development in *P. aeruginosa*, such as genetic mutations, horizontal gene transfer (HGT), and the interaction between virulence factors and resistance genes. It evaluates current detection methods, from traditional bacteriology to advanced molecular techniques, emphasizing the need for rapid and accurate diagnostics. This review also examines therapeutic strategies, including broad-spectrum antibiotics, novel drug candidates, combination therapies, and innovative approaches like RNA interference, CRISPR-Cas9 gene editing, and bridge RNA-guided gene editing. Importantly, this review highlights the distribution, migration, and environmental risks of MDR *P. aeruginosa*, underscoring its adaptability to diverse environments. It concludes by stressing the necessity for continued research and development in antimicrobial resistance, advocating for an integrated approach that combines genomics, clinical practice, and environmental considerations to devise innovative solutions and preserve antibiotic efficacy.

## 1. Introduction

*Pseudomonas aeruginosa* (*P. aeruginosa*) is a ubiquitous Gram-negative bacterium renowned for its remarkable adaptability to diverse environmental conditions [1]. This versatility, while advantageous in various applications, also renders *P. aeruginosa* a formidable opportunistic pathogen. It is particularly notorious for causing severe hospital-acquired infections [2], especially in immunocompromised patients, leading to a spectrum of life-threatening conditions including pneumonia, sepsis, and wound infections [3]. In recent years, the medical community has witnessed an alarming surge in multidrug resistance (MDR) of *P. aeruginosa* strains [4]. This bacterium’s exceptional ability to acquire and disseminate resistance mechanisms, such as β-lactamase production, outer membrane protein alterations, and efflux pump overexpression, has significantly complicated treatment strategies [5]. The rapid evolution of resistance, facilitated by horizontal gene transfer (HGT) and mutations, poses a substantial challenge to public health and medical safety.

The growing prevalence of MDR *P. aeruginosa* necessitates a multifaceted approach to combat this threat. This review aims to comprehensively analyze the mechanisms underlying MDR in *P. aeruginosa*, exploring its production, evolution, and spread. We critically evaluate current detection methods and their efficacy, and examine both established and emerging treatment strategies. We also elucidate the distribution, migration, and environmental risks of MDR *P. aeruginosa* in the environment. Furthermore, this review will assess innovative therapeutic avenues, including CRISPR-Cas9 or bridge RNA-guided gene editing techniques and the development of next-generation antibiotics. By addressing the challenges faced in clinical settings and evaluating the prospects of cutting-edge research, we aim to provide valuable insights for healthcare professionals, researchers, and policymakers in their collective effort to mitigate the impact of MDR *P. aeruginosa* on global health.

## 2. Understanding Multidrug-Resistance Mechanisms in *P. aeruginosa*

**Figure 1:**
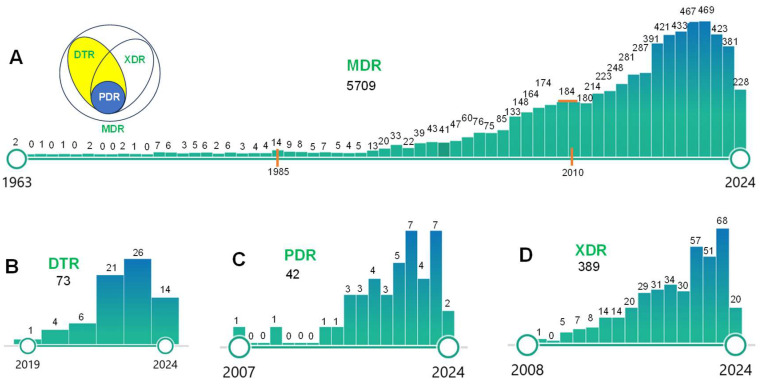
The data retrieved from the website (pubmed.ncbi.nlm.nih.gov) till July 20, 2024, showing the number of published papers related to MDR, DTR, PDR, and XDR of *P. aeruginosa*. (**A**) Keywords for “multidrug resistance MDR *Pseudomonas aeruginosa*”; the circle map indicates the schematic relationship among MDR, DTR, PDR, and XDR. (**B**) Keywords: “difficult-to-treat resistance DTR *Pseudomonas aeruginosa*”. (**C**) Keywords: “pan drug resistance PDR *Pseudomonas aeruginosa*”. (**D**) Keywords: “extensively drug-resistant XDR *Pseudomonas aeruginosa*”.

In the context of bacterial resistance, MDR, DTR (difficult-to-treat resistance), XDR (extensively drug-resistant), and PDR (pan drug-resistant) are terms used to describe the varying degrees of resistance that *P. aeruginosa* can exhibit towards antibiotics (Figure 1). MDR is a term used when *P. aeruginosa* is resistant to at least three different classes of antibiotics, which is a serious concern as it limits the options for the treatment of infections [6]. DTR refers to *P. aeruginosa* that are non-susceptible to a panel of first-line, high-efficacy, and low-toxicity antimicrobial agents [7]. This resistance complicates treatment strategies and necessitates the use of alternative, often less effective, antibiotics. XDR *P. aeruginosa* takes this a step further, being resistant to all but one or two categories of antimicrobials [8]. Infections caused by this type of *P. aeruginosa* are extremely difficult to treat, leading to high mortality rates. Finally, PDR *P. aeruginosa* is resistant to all agents in all antimicrobial categories, making infections caused by this type of *P. aeruginosa* untreatable with current drugs [9]. The emergence of PDR *P. aeruginosa* is a significant concern for global health. These terms help understand the severity of the resistance and make decisions about treatment strategies [10]. As shown in Figure 1, there are more publications for MDR *P. aeruginosa* than other types of *P. aeruginosa*, including DTR, XDR, and PDR. Thus, MDR is a major topic for bacterial resistance in *P. aeruginosa* [11], and we mainly review MDR *P. aeruginosa* in this study.

### 2.1. Main Factors Involved in MDR

**Figure 2:**
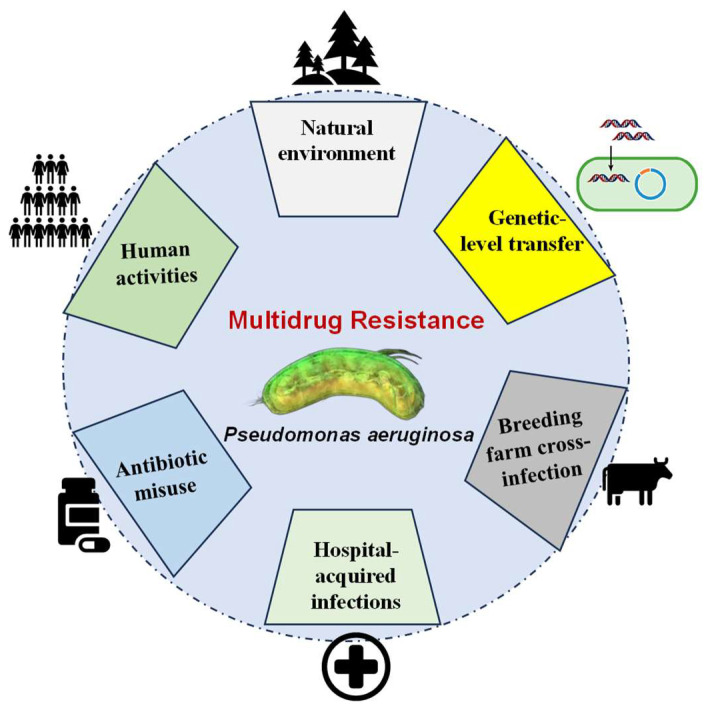
Interconnected drivers of multidrug resistance in *P. aeruginosa*. This schematic illustrates the synergistic interplay of environmental, anthropogenic, genetic, and behavioral factors underlying MDR emergence.

*P. aeruginosa*’s MDR stems from a confluence of factors, including environmental and anthropogenic influences on bacterial populations, rampant antibiotic misuse, cross-contamination in various settings, and genetic-level HGT (Figure 2).

**Figure 3:**
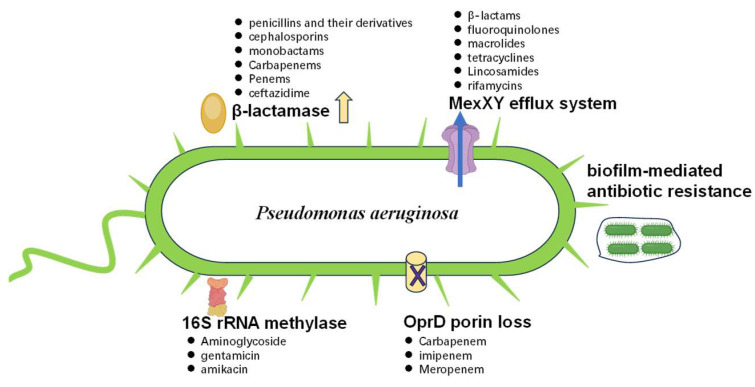
An illustrative depiction of the inherent mechanisms contributing to antibiotic resistance in *P. aeruginosa*. β-Lactamases are enzymes that hydrolyze the β-lactam ring of antibiotics, rendering them ineffective. MexXY are efflux pumps located on the bacterial cell membrane that actively transport antibiotics out of the cell, thereby reducing their intracellular concentration. A biofilm acts as a protective community of bacteria embedded in a self-produced matrix, which indicates reduced antibiotic penetration. The OprD protein is a porin channel in the outer membrane that, when down-regulated, restricts the entry of certain antibiotics. 16S rRNA methylase is an enzyme that modifies 16S rRNA, conferring resistance to aminoglycoside antibiotics by preventing their binding to the ribosome. The black dots on the chart represent antibiotics that are resistant.

The pathogen employs diverse antibiotic resistance mechanisms [12], such as the acquisition of resistance plasmids and integrons [13], adaptive mutations [14], active antibiotic efflux systems [15], and the production of drug-metabolizing enzymes [16]. Additionally, target site mutations further contribute to its formidable defense against antimicrobials [17]. The MDR phenotype in *P. aeruginosa* typically results from the synergistic action of these multiple resistance strategies (Figure 3).

Metallo-β-lactamases (blaVIM, blaIMP) and serine carbapenemases (blaKPC) are typically plasmid-encoded and associated with class 1 integrons. Genes, such as blaPER-1 and blaGES-5, are often located on IncP-6 plasmids, which facilitate their spread among Gram-negative pathogens. Target mutations (such as gyrA S83L and parC S87L) combined with the overexpression of efflux pumps (e.g., MexXY-OprM) reduce intracellular drug accumulation. Chromosomal mutations in pmrAB, phoPQ, and mgrB alter the lipid A structure, diminishing colistin binding. Although the plasmid-borne *mcr*-1 gene is rare in *P. aeruginosa*, its presence in clinical isolates raises concerns about HGT. This gene encodes a phosphoethanolamine transferase that modifies lipopolysaccharide, thereby conferring resistance. Consequently, addressing this critical issue demands a multifaceted research approach to develop comprehensive and effective prevention and control measures against this highly adaptable pathogen.

### 2.2. Improper Use of Antibiotics in Spreading MDR

The MDR of *P. aeruginosa* is closely related to the widespread and improper use of antibiotics. Excessive, misuse, or inappropriate use of antibiotics can lead to bacterial exposure to high concentrations of drugs, resulting in the development of resistance [18]. Furthermore, due to the high genetic plasticity and gene transfer ability of *P. aeruginosa*, they can transmit antibiotic resistance genes (ARGs) to other bacteria through HGT [19], leading to the rapid spread and dissemination of antibiotic resistance.

Improper antibiotic use can also affect the survival environment and ecosystem of *P. aeruginosa*. For example, antibiotic use may lead to an imbalance in the gut microbiome [20], allowing pathogenic bacteria like *P. aeruginosa* to more easily infect the host. Additionally, excessive use of antibiotics may also lead to an imbalance in the bacterial populations in hospital environments, facilitating the transmission and spread of resistant strains.

**Figure 4:**
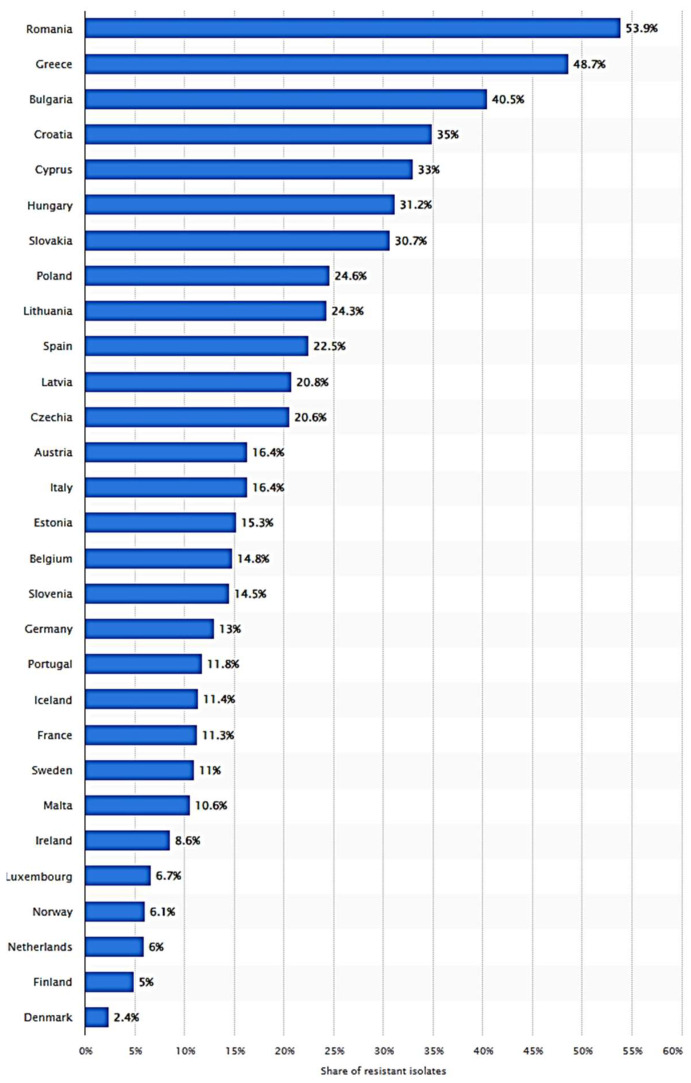
Percentage of invasive *P. aeruginosa* isolates resistant to carbapenem antibiotics in Europe in 2022, by country (data from Statista website, Available online: https://www.statista.com/statistics/1416724/share-of-invasive-p-aeruginosa-resistant-to-carbapenems-europe-by-country/ accessed on 10 April 2025).

The introduction of new antibiotics is often considered an effective measure against MDR bacteria. However, the misuse and irrational use of new antibiotics can also lead to the development of resistance [21]. First, the use of new antibiotics should be targeted, meaning that antibiotics should be selected based on the antibiotic susceptibility test results and the patient’s condition. In clinical practice, doctors sometimes overly rely on broad-spectrum or new antibiotics to treat difficult cases without conducting strict antibiotic susceptibility tests to determine the most appropriate treatment plan. Secondly, overuse of new antibiotics can accelerate the development of resistance [22]. New antibiotics are typically restricted in their use to avoid the development of resistance. However, in some countries, these restricted drugs are used extensively, accelerating the emergence of resistant strains. Furthermore, the improper use of new antibiotics can also lead to the development of resistant strains. For example, if new antibiotics are used incorrectly, such as premature discontinuation or inadequate dosage, it may lead to selective pressure for resistance genes, resulting in the development of resistance in the bacterial strain. Figure 4 shows invasive *P. aeruginosa* isolates resistant to carbapenem antibiotics in Europe in 2022. Romania ranks first for the highest percentage of resistance, which is alarming given the potential implications for public health and the effectiveness of current treatment strategies. It is crucial to implement antibiotic stewardship programs that promote the judicious use of antibiotics.

Therefore, to minimize the promotion of resistance by new antibiotics, it is necessary to develop reasonable clinical application guidelines and regulations, and strengthen training and education for healthcare professionals to ensure the correct and rational use of new antibiotics [23]. Additionally, during the development of new antibiotics, the risks of misuse and inappropriate use should be considered to maximize the delay in the development of bacterial resistance to antibiotics [24].

Therefore, to effectively prevent and control MDR in *P. aeruginosa*, it is necessary to adopt rational antibiotic use strategies [25]. This includes strictly controlling the prescription and use of antibiotics, adhering to antibiotic use guidelines and regulations, and implementing effective infection control measures in healthcare facilities. Simultaneously, it is essential to strengthen public and healthcare professional education and awareness regarding antibiotic use and MDR to reduce unnecessary and improper antibiotic use [26].

## 3. The Contribution of Evolutionary Dynamics in Shaping Resistance

*P. aeruginosa* has various metabolic pathways and adaptive abilities, enabling it to survive and proliferate in different environments. However, in humans and animals, *P. aeruginosa* often causes various infections, especially in patients with compromised immune function, such as those with leukemia, burns, or in the perioperative period. *P. aeruginosa* has high adaptability and pathogenicity, making it a major pathogen for hospital-acquired and nosocomial infections [27].

The evolution of MDR in *P. aeruginosa* is a complex process involving the interplay of multiple factors. In *P. aeruginosa*, the formation of MDR is mainly due to changes at the genetic level, including gene mutations, HGT, and gene rearrangements [28]. Furthermore, the MDR of *P. aeruginosa* is closely related to its survival environment, such as the widespread use of antibiotics and disinfectants in hospitals and farms, which have had a significant impact on the development of antibiotic resistance in *P. aeruginosa*. The evolution of MDR in *P. aeruginosa* is also closely related to changes in its pathogenicity [29]. During the pathogenic process of *P. aeruginosa*, some virulence factors are also involved in the production and evolution of MDR. For example, the outer membrane proteins and inner membrane proteins of *P. aeruginosa* not only participate in antibiotic efflux and metabolism but also protect cells from the toxicity of compounds. Furthermore, some regulatory genes are also involved in the regulation of MDR in *P. aeruginosa*, such as MexAB-OprM, MexXY-OprM, and MexCD-OprJ [30].

The evolution of MDR in *P. aeruginosa* is an ongoing process of development and evolution [31]. In the future, it is necessary to further investigate the genes and regulatory mechanisms related to MDR to develop new treatment strategies and prevention and control measures.

### 3.1. Co-Evolution of Virulence Factors and ARGs

Virulence factors and antibiotic-resistance genes are often located on the same transferable plasmids or integrons, and the co-evolution of these genes may lead to an increase in toxicity and antibiotic resistance as *P. aeruginosa* continues to evolve. Some studies have shown that multiple virulence factors and ARGs on the plasmids and integrons of *P. aeruginosa* may be co-regulated [32]. The co-regulation of these genes may be related to the environmental adaptability of bacteria, as they may be expressed in various environments, including those within humans and animals.

The co-evolution of virulence factors and ARGs in *P. aeruginosa* may be an important mechanism for the evolution of MDR. Virulence factors and ARGs in *P. aeruginosa* are often encoded on the same DNA segment [27,33], which may lead to their co-evolution. Within bacterial cells, antibiotics typically induce DNA recombination processes, leading to the cross-transfer of virulence genes and ARGs. Additionally, plasmids in *P. aeruginosa* may play a crucial role, as they can transfer multiple drug resistance genes between different strains.

The genes and gene regulatory mechanisms related to MDR are also an important aspect of the evolution of MDR in *P. aeruginosa*. Many MDR-related genes have been discovered in *P. aeruginosa*, including those encoding drug efflux pumps, modifying enzymes, and target sites [34]. These genes can be regulated through various mechanisms, such as global regulation, two-component systems, transcription factors, and non-coding RNAs.

In summary, the MDR of *P. aeruginosa* is the result of the combined action of various mechanisms. Understanding these mechanisms is crucial for developing effective prevention and treatment strategies against *P. aeruginosa*.

### 3.2. MDR-Related Genes and Gene Regulatory Mechanisms

The development of MDR in *P. aeruginosa* is closely related to the regulatory mechanisms of its resistance genes. Studies have shown that other genes regulate the expression of some resistance genes. For example, research has demonstrated that the expression of the *qnrVC1* and *qnrVC2* genes in *P. aeruginosa* is regulated by the crp (cAMP receptor protein) gene and qnrVC1 and qnrVC2 are frequently integrated into class 1 integrons [35]. These integrons are frequently located on broad-host-range plasmids, such as those belonging to the IncP-6 and IncP-7 replicon types, which are particularly prevalent in *P. aeruginosa* and other Gram-negative pathogens. While the crp gene itself is regulated by the hirA (hemolysin information regulator A) gene. Additionally, some studies have also shown that certain ARGs can spread between different bacterial strains through HGT [36], which plays an important role in the rapid dissemination of antibiotic resistance and the acquisition of new resistance genes. Therefore, the MDR of *P. aeruginosa* is a complex issue involving the interaction of multiple factors. In-depth research on the evolution and regulatory mechanisms of its resistance is crucial for a better understanding of antibiotic resistance and the development of prevention and treatment strategies.

## 4. MDR Detection Methods in *P. aeruginosa*

### 4.1. Traditional Bacteriological Detection Methods

Traditional bacteriological detection methods for *P. aeruginosa* mainly include colony counting, bacterial morphological characteristics, physiological and biochemical properties, and antimicrobial susceptibility testing [37,38,39]. These methods require culturing and propagating the bacterial strains, followed by antimicrobial susceptibility testing to determine the sensitivity of the bacteria to different antibiotics. Traditional bacteriological detection methods have advantages, such as reliability and stable results, but they are time-consuming, often requiring several days to weeks to obtain results. Additionally, these methods may produce false-negative or false-positive results and may not detect some new resistant strains. Therefore, in the detection of MDR in *P. aeruginosa*, traditional bacteriological detection methods have gradually been replaced by other more advanced detection methods.

To improve detection efficiency and accuracy, many new detection methods have been developed. These methods include molecular biology detection methods and rapid diagnostic methods [40]. These new methods can rapidly and accurately detect MDR in *P. aeruginosa* and have significant clinical implications.

### 4.2. Molecular Biology Detection Methods

Molecular biology detection methods are rapid, accurate, and highly sensitive methods that have become indispensable for the detection of MDR in *P. aeruginosa* [41]. These methods primarily detect genes or gene mutations related to antibiotic resistance in *P. aeruginosa*.

Common molecular biology detection methods include polymerase chain reaction (PCR), real-time fluorescent quantitative PCR (qRT-PCR), DNA microarray technology, and next-generation sequencing technologies. Among these, PCR is one of the most commonly used methods [39], which involves amplifying specific gene fragments of *P. aeruginosa* [42], such as β-lactamase genes, aminoglycoside resistance genes, and phosphoacetyl transferase genes. qRT-PCR has the advantages of a high sensitivity, high specificity, and rapid detection, allowing for the simultaneous detection of multiple antibiotic resistance-related genes within hours. DNA microarray technology can simultaneously detect multiple antibiotic resistance-related genes and virulence factor genes, but it is relatively expensive. Next-generation sequencing technology has the advantages of a high throughput and high resolution [43,44], enabling comprehensive analysis of the genome information and expression profiles of *P. aeruginosa*, providing a powerful tool for in-depth understanding of the mechanisms and evolution of MDR in *P. aeruginosa*.

Recent advances in the molecular detection of *P. aeruginosa* include RPA-CRISPR-Cas12a [45] and LAMP-CRISPR-Cas12a [46], which offer high sensitivity and specificity with minimal sample preparation, quick detection, and applications in point-of-care testing (POCT). Other techniques like digital droplet PCR [47] and biosensors [41] are also emerging, promising faster, more accurate, and cost-effective diagnostics. These innovations are set to transform clinical diagnostics and infection control, aiding in the fight against multi-drug resistance.

Overall, molecular biology detection methods can rapidly and accurately detect MDR in *P. aeruginosa*, but they require pre-treatment steps, such as DNA extraction, and have high demands on experimental techniques and quality control. In the future, with the continuous development of new technologies, detection methods for MDR in *P. aeruginosa* will become faster, more accurate, more convenient, and less expensive.

## 5. Treatment Strategies for MDR in *P. aeruginosa*

### 5.1. Current Treatment Strategies

Currently, the treatment of MDR *P. aeruginosa* infections primarily relies on the use of antibiotics to control the infection. However, due to the MDR of this bacterial species, the effectiveness of traditional antibiotic treatment has been limited [48]. Some broad-spectrum antibiotics (such as carbapenems, aminoglycosides, etc.) can still be used to treat *P. aeruginosa* infections, but for certain highly resistant strains, their therapeutic effects are not ideal. Furthermore, overuse of broad-spectrum antibiotics may further exacerbate bacterial resistance.

In addition to broad-spectrum antibiotics, some new drugs for treating *P. aeruginosa* infections are being developed [49], such as levofloxacin, ceftolozane/tazobactam, and telavancin. Although these drugs are still in the early stages of clinical application research, they may become alternative treatment options for *P. aeruginosa* infections in the future. Furthermore, antibiotic combination therapy is also widely used in the treatment of MDR bacterial infections [50], including *P. aeruginosa* infections. The combination of different antibiotics can produce synergistic effects and enhance antimicrobial activity.

In summary, current strategies for treating MDR *P. aeruginosa* infections include the use of broad-spectrum antibiotics, the development of new drugs, and antibiotic combination therapy. As research on this bacterial species continues, more effective treatment strategies will be developed.

### 5.2. New Treatment Strategies and Drugs

In the treatment of MDR in *P. aeruginosa*, new strategies and drugs are continuously being researched and developed. One important research direction is antibiotics targeting the β-lactamases produced by *P. aeruginosa*. Currently, some new antibiotics have shown promising antimicrobial effects in clinical trials. For example, Ceftazidime-Avibactam is a new β-lactamase inhibitor combined with an antibiotic [51]. Its inhibitor, Avibactam, can inhibit the activity of various β-lactamases, effectively preventing the development of antibiotic resistance in bacteria. This drug has been approved by the U.S. Food and Drug Administration (FDA) for the treatment of complicated urinary tract infections and intra-abdominal infections and has shown good therapeutic effects in clinical trials. Plazomicin is a new aminoglycoside antibiotic with broad-spectrum antimicrobial activity and good bactericidal effects against various MDR strains [52], including *P. aeruginosa*. This drug has been approved by the FDA for the treatment of complicated urinary tract infections and intra-abdominal infections and has shown good antimicrobial effects in clinical trials. Furthermore, some studies have also shown that immunotherapy strategies using humanized monoclonal antibodies targeting MDR in *P. aeruginosa* are feasible [53,54]. Colistin resistance mediated by mobile colistin resistance 1 gene (*mcr*-1) poses a significant threat due to its plasmid-borne nature and potential for dissemination. Surveillance for *mcr*-1 in *P. aeruginosa* is critical, as its presence may necessitate alternative therapies, such as novel β-lactam/β-lactamase inhibitors (e.g., cefiderocol) or phage-antibiotic combinations. Moule et al. reported the peptide-mimetic treatment of *P. aeruginosa* using a mouse model [55], indicating that peptide-mimetic TM5 may be an alternative therapy for MDR *P. aeruginosa* respiratory infections.

Recent advances in phage therapy have shown promising results for treating *P. aeruginosa* infections [56], particularly in cases of antibiotic resistance. Researchers are exploring novel approaches to enhance phage efficacy and overcome bacterial defense mechanisms. These strategies include using phage cocktails to target multiple bacterial strains simultaneously [57], engineering phages to improve their stability and host range, and combining phages with traditional antibiotics for synergistic effects [58]. Additionally, personalized phage therapy, where specific phages are selected based on the patient’s bacterial isolate, is gaining traction [59]. Nanoparticle-based delivery systems are being developed to improve phage distribution and persistence in the body. Furthermore, scientists are investigating the potential of phage-derived enzymes, such as endolysins [60], as standalone antimicrobial agents. These innovative approaches aim to harness the full potential of phages in combating *P. aeruginosa* infections, offering hope for patients with limited treatment options.

In general, although the treatment of MDR in *P. aeruginosa* remains challenging, the research progress on new treatment strategies and drugs provides new hope for its treatment. In the future, these new strategies and drugs will need further clinical research and validation to better address the challenges of MDR in *P. aeruginosa*.

### 5.3. Antibiotic Combination Therapy

With the continuous emergence of MDR strains of *P. aeruginosa*, single-antibiotic treatment has become increasingly difficult to effectively control infections. Therefore, antibiotic combination therapy has become an effective treatment choice. Combination therapy can utilize the different mechanisms of action of different antibiotics to achieve synergistic or enhanced therapeutic effects, increasing the success rate of treatment.

Some existing studies have shown that antibiotic combination therapy can effectively treat *P. aeruginosa* infections. Additionally, the combination of fluoroquinolone antibiotics with β-lactam antibiotics can also achieve good therapeutic effects [61]. When researching antibiotic combination therapy, factors, such as antibiotic interactions, dosages, and routes of administration need to be considered. At the same time, it is important to note that the combined use of antibiotics may increase the occurrence of adverse reactions and drug intolerance. Therefore, selecting appropriate antibiotic combinations and dosages, as well as controlling the duration and frequency of use, are crucial for improving the efficacy and safety of combination therapy.

### 5.4. Treatment Methods Targeting Resistance Mechanisms

The MDR of *P. aeruginosa* is mainly due to its numerous resistance genes and regulatory mechanisms, and traditional antibiotic treatment has been unable to effectively control its infections. Therefore, researchers have begun to explore treatment methods targeting resistance mechanisms.

One feasible treatment method is to use RNA interference technology to inhibit the expression of resistance genes. Research has found that using RNA interference technology to inhibit the expression of resistance genes [62]. Additionally, new antibiotics that inhibit the β-lactamases of *P. aeruginosa* are also under research. Another feasible treatment method is to use CRISPR-Cas9 technology [63] or bridge RNA-guided gene editing [64] to excise resistance genes. Research has shown that using CRISPR-Cas9 technology to successfully excise resistance genes [65], such as *blaIMP-1* and *blaNDM-1* in *P. aeruginosa* can effectively enhance the antimicrobial activity of various antibiotics. The bridge RNA-guided gene editing technique, a novel approach introduced in 2024 [64,66], offers a unique method for treating *P. aeruginosa* infections. This technique employs a special ‘bridge’ RNA molecule with two internal loops. These loops are designed to base-pair with both the target DNA and the donor DNA, which is the IS*110* element. The loops can be independently reprogrammed, enabling sequence-specific recombination between two DNA molecules. This allows for DNA insertion, excision, and inversion at specific genomic sites. The IS*110* bridge system broadens the scope of nucleic acid-guided systems, providing a comprehensive mechanism for genome design [67].

Targeting resistance mechanisms in *P. aeruginosa* MDR is a popular research area, and researchers are continuously exploring new treatment strategies. In the future, it is necessary to conduct more in-depth research on the resistance mechanisms of *P. aeruginosa* to discover more new treatment methods.

## 6. Distribution, Migration, and Risks of MDR *P. aeruginosa* in the Environment

**Figure 5:**
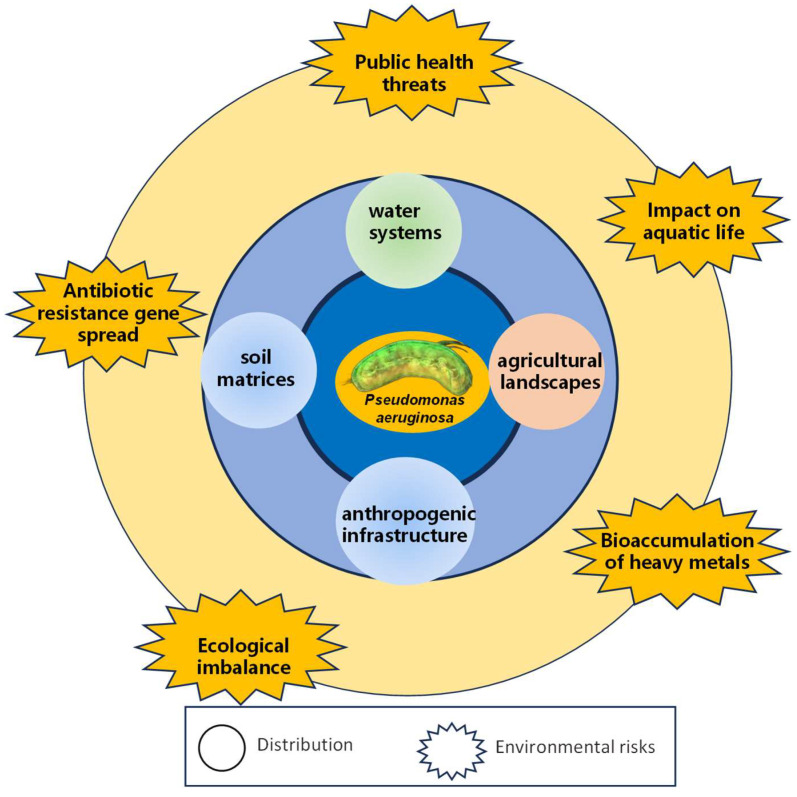
The distribution and environmental risks of MDR *P. aeruginosa*. This diagram illustrates the complex interactions and environmental pathways through which MDR *P. aeruginosa* spreads and poses risks.

The emergence and proliferation of MDR *P. aeruginosa* represent a critical global health and environmental challenge that transcends traditional clinical boundaries, revealing a complex ecological phenomenon with profound implications for human, animal, and environmental health. This opportunistic pathogen demonstrates remarkable adaptability, navigating and colonizing diverse environmental niches through sophisticated genetic mechanisms that enable survival across heterogeneous ecosystems. The distribution of MDR *P. aeruginosa* is characterized by a sophisticated interplay of multiple reservoirs [68], including water systems, soil matrices, agricultural landscapes, and anthropogenic infrastructure (Figure 5), where the bacterium leverages extraordinary genetic plasticity to develop and maintain resistance through HGT, plasmid exchange, and complex mutational strategies.

Aquatic environments, particularly wastewater treatment facilities, municipal water networks, and natural water bodies, emerge as pivotal transmission conduits, providing optimal conditions for bacterial survival through nutrient availability, temperature variability, and intricate microbial interactions that facilitate genetic recombination and adaptive evolution. The migration of MDR *P. aeruginosa* is further amplified by human activities [69], industrial processes, and global transportation networks, with anthropogenic interventions, such as improper waste management, inadequate sanitation infrastructure, and transboundary movement of goods creating sophisticated bacterial dispersion mechanisms. Agricultural ecosystems play a significant role in propagating these resistant strains [70], with intensive farming practices and extensive antibiotic utilization in livestock management generating conducive conditions for bacterial adaptation, while soil microbiomes act as complex genetic reservoirs where continuous genetic material exchange promotes the emergence of novel resistance mechanisms. Climate change and environmental transformations additionally influence bacterial migration patterns, with alterations in temperature, precipitation, and ecological dynamics potentially enhancing bacterial adaptability and creating more expansive and resilient ecological niches. The environmental risks associated with MDR *P. aeruginosa* extend beyond immediate transmission concerns, presenting significant challenges to ecosystem stability and human health protection through the potential transfer of resistance genes between bacterial species and the ability of these genetic elements to persist in environmental matrices (Figure 5). The bacterium’s capacity to form sophisticated biofilm structures enhances its environmental resilience, allowing it to shield bacterial populations from stressors and facilitate genetic exchange through conjugative processes, with urban wastewater treatment infrastructures, agricultural irrigation systems, and interconnected hydrological networks serving as primary conduits for widespread distribution of resistant genetic elements across geographical boundaries [71]. Technological advancements in genomic surveillance and molecular epidemiology have revolutionized our understanding of these distribution dynamics, enabling precise tracking of bacterial lineages and resistance gene evolution through whole-genome sequencing and advanced bioinformatics approaches that reveal intricate genetic networks demonstrating how resistance mechanisms represent complex, interconnected ecological processes with global implications.

## 7. Conclusions

Multidrug-resistant *P. aeruginosa* poses a significant public health threat and environmental risks, arising from complex interactions of antibiotic misuse, genetic changes, and environmental factors. This opportunistic pathogen’s widespread resistance compromises treatment efficacy, necessitating innovative strategies. Addressing this challenge requires a comprehensive approach integrating genomics, molecular biology, and environmental studies. Current detection methods range from traditional to cutting-edge techniques, while therapeutic strategies focus on combination therapies and novel antimicrobials. Future research should leverage genomic and molecular insights to develop targeted interventions, including new antibiotic classes and resistance-neutralizing strategies. Global surveillance and interdisciplinary collaboration are essential in tackling this multifaceted issue. Ultimately, combating *P. aeruginosa*’s MDR, especially in the environment, demands a concerted effort combining advanced research, prudent clinical practices, and comprehensive environmental risk measures to protect our antimicrobial arsenal for future generations.

## Data Availability

No new data were created or analyzed in this study. Data sharing is not applicable to this article.
